# Using machine learning improves predictions of herd-level bovine tuberculosis breakdowns in Great Britain

**DOI:** 10.1038/s41598-021-81716-4

**Published:** 2021-01-26

**Authors:** K. Stański, S. Lycett, T. Porphyre, B. M. de C. Bronsvoort

**Affiliations:** 1 The Epidemiology, Economics and Risk Assessment (EERA) Group, The Roslin Institute, Royal (Dick) School of Veterinary Studies, The University of Edinburgh, Easter Bush, Midlothian, UK; 2 Université Lyon 1, CNRS, VetAgro Sup, Laboratoire de Biométrie Et Biologie Evolutive, Université de Lyon, Villeurbanne Cedex, France

**Keywords:** Machine learning, Tuberculosis, Risk factors, Epidemiology

## Abstract

In the United Kingdom, despite decades of control efforts, bovine tuberculosis (bTB) has not been controlled and currently costs ~ £100 m annually. Critical in the failure of control efforts has been the lack of a sufficiently sensitive diagnostic test. Here we use machine learning (ML) to predict herd-level bTB breakdowns in Great Britain (GB) with the aim of improving herd-level diagnostic sensitivity. The results of routinely-collected herd-level tests were correlated with risk factor data. Four ML methods were independently trained with data from 2012–2014 including ~ 4700 positive herd-level test results annually. The best model’s performance was compared to the observed sensitivity and specificity of the herd-level test calculated on the 2015 data resulting in an increased herd-level sensitivity from 61.3 to 67.6% (95% confidence interval (CI): 66.4–68.8%) and herd-level specificity from 90.5 to 92.3% (95% CI: 91.6–93.1%). This approach can improve predictive capability for herd-level bTB and support disease control.

## Introduction

Bovine tuberculosis (bTB) is a chronic, infectious respiratory disease of cattle caused by *Mycobacterium bovis*. Infection can be clinically inapparent but animals can shed bacteria in milk leading to risk of human infections and which was the original driver for control of bTB in Great Britain (GB). The majority of milk produced in GB is pasteurized, thereby drastically limiting the risk of infecting humans from the consumption of milk and dairy products. However, the risk of bTB spread still poses limitations for international trade of live animals and animal products. In GB, the number of herds with bTB increased significantly in 2002 largely due to less stringent surveillance and control measures during the foot and mouth disease (FMD) outbreak of 2001^1^. Since then, and despite more than 15 years with an intensive testing regime and removal of infected animals, the disease prevalence has been progressively increasing in England and Wales^2^, and currently involved around 40% of cattle herds in the high risk area of infection. In contrast, Scotland is considered a bTB-free country, where infected animals have rarely been detected since 1995^3^. bTB has a negative impact on cattle productivity, trade on a national and international level and the disease control costs are estimated at £100 million annually^4^.

The single intra-dermal comparative cervical tuberculin (SICCT) test is currently the primary screening test for bTB herd surveillance in GB. The procedure involves injecting tuberculin, which is a purified protein derivative (PPD) extracted from *M. bovis* cultures, into the skin of an animal and measuring the swelling of the area caused by a delayed type hypersensitivity response to the PPD. Karolemeas et al*.*^5^ estimated the sensitivity (Se) of the animal-level SICCT test at 70–89%, whereas Goodchild et al.^6^ estimated the specificity (Sp) of the test to be 99.98%. The results of all animals tested within a herd at a given time are aggregated to give a herd-level SICCT result, as opposed to animal-level results. If at least one animal in a herd is found to be a reactor, the herd-level SICCT test is considered positive and cattle movement restrictions are imposed upon this herd. The reactor animals are culled and their carcasses are subject to lesion inspection at abattoirs. However, lesion inspection procedures are known to be insensitive^7^ and visible lesions are found in only 30–45% of reactors in GB^8^.

The probability of herd-level bTB breakdowns is dependent on herd characteristics and management practices, both of which may influence the infection risk. Gates et al.^9^ reported that between-farm cattle movement is one of the main causes of spread of bTB. For this reason, cattle movement restrictions are imposed according to the results of routine SICCT tests. Due to the imperfect Se of the SICCT test, chances that infected cattle may move undetected from farm to farm still remain. As such, farms purchasing large number of cattle may therefore be at greater risk of becoming infected with bTB. Furthermore, increasing herd size has been found associated with a higher risk of bTB infection in various production systems^10,11^ and has been shown as key component in the within-herd bTB transmission dynamics^12^. Finally, *M. bovis* can infect mammals other than cattle, which, presents a major difficulty in eradicating the disease. It can remain in wildlife reservoirs and re-infect cattle herds^13^. Populations of many species can act as such reservoirs, e.g. white-tailed deer in the USA^14^, brush-tail possum in New Zealand^15^ and badgers in the UK^16^. Proximity to badger sets has been linked to increased bTB risk in UK and badgers have been culled in an attempt to limit the impact of this transmission route.

Machine learning (ML) modelling refers to statistical modelling techniques which detect patterns within data, as opposed to process-driven models which rely on understanding of system dynamics. ML algorithms make predictions by exploiting correlations between input variables (features) and an output variable (target), while being robust to inter-correlation within the set of input variables^17^. Employing such techniques can potentially improve the accuracy of herd-level bTB diagnosis by including data which may influence bTB breakdown risk, e.g. cattle movements^9^ and herd size^12^ in the analysis and in the interpretation of the herd bTB status.

In a previous study on the application of ML methods to GB cattle data, Ortiz-Pelaez and Pfeiffer^18^ developed a model to predict the risk of disease presence in Welsh cattle herds. This study was not bTB specific and a herd was considered positive if any of the most frequent infectious disease of cattle in GB (including bTB) was diagnosed in at least one animal. The ML methods were shown to accurately predict herd-level infection risk. The selection of variables which were particularly important for the risk prediction, was in agreement with previous studies, i.e. herd size, number of cattle movements and holding type. The study shows that risk factors can be quantified and combined to provide a single measure of herd-level infection risk. In the context of bTB control programme, however, risk factors would ideally be collated with the results of routine herd-level SICCT tests. This could inform interpretation of the herd-level SICCT test and improve diagnostic sensitivity.

Records of results of individual animal SICCT tests were analysed by Adamskiy et al*.*^19^. They used the ML methodology to improve the accuracy of the SICCT test. All the data were taken from the VetNet database whose features and purpose are described in the National Statistics report *Background and methodology to the National Statistics on the Incidence of Tuberculosis (TB) in Cattle in Great Britain*^20^. VetNet contains only the records of the SICCT test results which has been positive. Post-mortem *M. bovis* culture or the gamma-interferon test follow each positive SICCT test in order to confirm it and their results are also recorded in the database. The study showed that a ML interpretation of a positive SICCT test is capable of correctly detecting false positive results. Adamskiy et al*.* reported that two thirds of false positives can be identified at the cost of misclassifying 10% of true positives. It is important to note that the delay of the *M. bovis* culture test has not been considered and it was assumed that the result was available immediately. While such a result may represent the base for improving bTB diagnostics, it only leads to an increase in the specificity of bTB diagnostics. The current bTB control program in GB is, however, limited by the imperfect sensitivity of the SICCT test, which can result in undetected infected herds. To increase the sensitivity of bTB diagnostics, a ML algorithm has to be presented with both positive and negative SICCT test results. In this way, the model can learn to identify false negatives.

In this study, we used the new bTB surveillance database (SAM), which replaced the VetNet database since 2011 and includes positive and negative SICCT results, to develop a ML model which could accurately predict future herd-level bTB breakdowns in GB. To do so, we incorporated past and current SICCT test results and collated them with data directly and indirectly related to bTB infection risk. The model was evaluated as a means to increase the herd-level sensitivity (HSe) and specificity (HSp) of the bTB diagnostic testing by providing a more sophisticated interpretation of the herd-level SICCT test results.

## Results

In this study, the results of animal-level SICCT tests conducted in GB between 2012 and 2015 were aggregated and dichotomised at the herd-level, informing whether at least one animal had tested positive in a herd. In total, over 500,000 herd-level results were recorded during the study period. These records were then extended with risk factor data of the farms and used as input to ML models to predict future herd-level bTB breakdowns.

### Case definition

In order to develop predictive models, both ground truth positives (future bTB breakdowns) and negatives (bTB-free farms) had to be defined to train and evaluate the ML algorithms. As such, a herd was defined as a future breakdown if a bTB infection of at least one animal was confirmed by lesion inspection or *M. bovis* culture within 90 days following the SICCT test.

### Summary statistics of the SICCT performance

To provide a measure of performance of the SICCT test, which will be used as a baseline to compare against a ML predictive model, we calculated the observed HSe and HSp of the herd-level SICCT test separately for data in every year from 2012 to 2015 according to the definition of future bTB breakdown in this study. The HSe and HSp of SICCT stratified by year are shown in Table 1.

**Table 1:** Performance of the herd-level SICCT test by year.

Year	No of positives^a^	No of negatives^b^	HSe in % (95% CI)^c^	HSp in % (95% CI)^d^
2012	4693	117,431	63.8 (62.5–65.1)	89.2 (88.3–90.1)
2013	4605	119,514	61.9 (60.4–63.5)	90.0 (89.0–90.9)
2014	4818	123,870	60.8 (59.3–62.3)	91.2 (90.4–92.1)
2015	4686	126,167	61.3 (60.0–62.7)	90.5 (89.6–91.3)

^a^Number of ground truth positives.

^b^Number of ground truth negatives.

^c^Observed sensitivity of the SICCT test (95% confidence interval).

^d^Observed specificity of the SICCT test (95% confidence interval); Confidence intervals were calculated using a one-sample binomial proportion asymptotic approximation.

### Machine learning predictive performance

The dataset used in this study consisted of collated data from different sources: SICCT test results of a herd, past bTB breakdowns, cattle movements, land cover and climate data. From these data we derived 139 variables characterising herds, including current SICCT test results. These variables were then combined into vectors, such that every herd at the time of a SICCT test was represented as a vector. The vectors were fed into ML algorithms as input. The target output value of models was the 0 or 1 indication of a future bTB breakdown (where 1 corresponds to breakdown and 0 corresponds to no breakdown). The whole dataset was split into three disjoint subsets. A training set consisted of data from 2012 to 2013, totalling 9300 ground truth positives and 236,998 negatives. It was used to optimise the internal parameters of ML methods in the training phase. A development set included data from 2014, where 4819 data points were positive and 123,949 were negative. This subset was used for initial evaluation to optimise the settings of ML algorithms and to select the best-performing model. A testing set of 2015 data consisted of 4690 ground truth positive herds and 126,255 negatives. A final evaluation of the best-performing model was conducted on the testing set and the performance of this model was compared to the observed HSe and HSp of the SICCT test calculated for the same data points.

**Figure 1:**
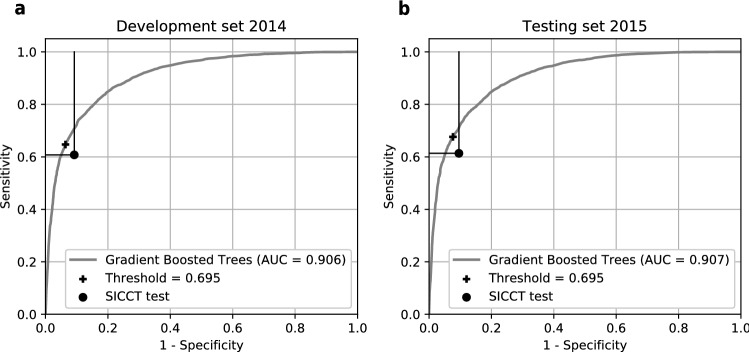
Receiver operating characteristic curves of Gradient Boosted Trees performing on the development and testing datasets. Dots represent the HSe and HSp of the herd-level SICCT test observed in the development and testing sets. Crosses show the HSe and HSp of GBT predictions dichotomized with a threshold of 0.695.

It is impossible to know upfront which ML algorithm will provide the most accurate predictions for a specific problem. Therefore, we chose four commonly used ML methods—Support Vector Classifier (SVC), Neural Network (NNET), Random Forest (RF) and Gradient Boosted Trees (GBT)—and independently trained them on the training set. Their performance on the development set was measured and compared to select the best performing model based on the area under the receiver operating characteristic curve (AUC) of their predictions. SVC, NNET, RF and GBT achieved an AUC of 0.875, 0.899, 0.904 and 0.906, respectively. As a result, GBT was selected as the best-performing method and used for further analysis. The model obtained AUC of 0.907 on the testing set of data from 2015 that was new to the model, which is consistent with its performance on the development set. A portion of the curve shown in Fig. 1 reaches beyond the HSe and HSp of the herd-level SICCT test as calculated for the development and testing datasets. The decision threshold, used to dichotomize the model’s output, was optimized to be in the middle of this portion for the development set. This resulted in the threshold value of 0.695, i.e. all breakdown probability estimates above 0.695 were classified as positives. The value of the threshold was transferable to the testing data and it led to improvement of both HSe and HSp.

In order to obtain confidence intervals of HSe and HSp, and to test the statistical significance of the difference between the observed SICCT test results and GBT’s predictions, HSe and HSp were independently calculated in 100 disjoint subsets of the testing set. These subsets were randomly sampled without replacement. Then, a Student t-test was applied to the HSe and HSp distributions. On the testing 2015 data, the herd-level SICCT test alone obtained a mean HSe of 61.3% (95% CI: 60.0–62.7%) and HSp of 90.5% (95% CI: 89.6–91.3%). GBT achieved a HSe of 67.6% (95% CI: 66.4–68.8%, an increase of 6.3%-points, *p*-value < 0.0001 paired sample two-tailed Student’s t-test) and HSp of 92.3% (95% CI: 91.6–93.1%, an increase of 1.8%-points, *p*-value < 0.0001 paired sample two-tailed Student’s t-test). The model was capable of predicting future bTB breakdowns (within the next 90 days post testing) which were undetected by the SICCT test, i.e. ground truth positives which were identified by GBT, but tested negative in SICCT test. In 2015 data, GBT correctly predicted 297 breakdowns in addition to 2874 breakdowns detected by the herd-level SICCT test alone. This is a 10.3% improvement of detection of bTB infected herds.

### Spatial distribution

The distribution of true positives (TP) and false positives (FP) in GB regions is shown in Table 2. The table presents the summary statistics of herd-level SICCT test results and GBT predictions, and compares them to the total number of ground truth positives and negatives. The difference column highlights in which region GBT performed better or worse than the SICCT. Here, we treat positive values of TP difference and negative values of FP difference as improvements. Increased TPs were apparent only in Wales, West England and North England. A decrease in the number of FPs, however, was present across all regions. The performance of GBT was compared to SICCT results also using positive predictive values (PPV) and negative predictive values (NPV) in the whole GB and regions (Table 2). PPVs were increased in all regions, but NPVs improved only in Wales, West England and North England.

**Table 2:** True positives and false positives in 2015 testing set by region.

Statistic	Region	GBT^a^	SICCT^b^	Difference^c^	Pos^d^	Neg^e^	GBT PV (%)^f^	SICCT PV (%)^g^
TP	Wales	482	477	5	731	31,923	20.20	15.20
	West E	1997	1754	243	2897	46,208	26.64	21.61
	East E	143	153	− 10	242	16,990	15.96	12.92
	North E	542	482	60	803	27,868	28.72	22.93
	Scotland	7	8	− 1	13	3178	10.61	6.20
	GB	3171	2874	297	4686	126,167	24.91	19.59
FP	Wales	1904	2661	− 757	731	31,923	99.18	99.14
	West E	5500	6362	− 862	2897	46,208	97.84	97.21
	East E	753	1031	− 278	242	16,990	99.39	99.45
	North E	1345	1620	− 275	803	27,868	99.03	98.79
	Scotland	59	121	− 62	13	3178	99.81	99.84
	GB	9561	11,795	− 2234	4686	126,167	98.72	98.44

^a^Summary statistics of GBT predictions on the testing set.

^b^Summary statistics of herd-level SICCT test results on the testing set.

^c^Difference of GBT and SICCT.

^d^The total number of ground truth positives in the testing set.

^e^The total number of ground truth negatives in the testing set.

^f^Predictive values of GBT predictions. The first six lines for positive predictive values (PPV) and the last six lines for negative predictive values (NPV).

^g^Predictive values of SICCT predictions. The first six lines for positive predictive values (PPV) and the last six lines for negative predictive values (NPV).

**Figure 2:**
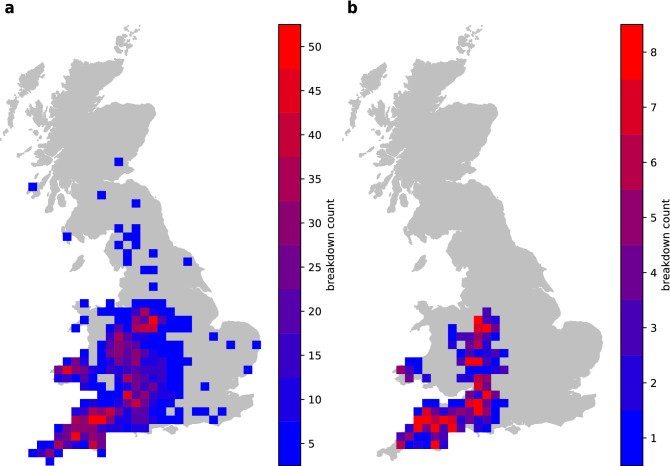
(**a**) Map of all 3171 bTB infected farms identified by the GBT model in the testing set of 2015 data. (**b**) Map of 297 infected farms which were undetected by the herd-level SICCT test, but identified by the GBT model in the testing set of 2015 data. The maps were drawn using the Matplotlib v2.1.0 library of Python^21^.

The maps of spatial distribution of TP presented in Fig. 2 show that the increase in TP offered by the model is concentrated in the region of high bTB breakdown frequency.

### Input variable importance

**Figure 3:**
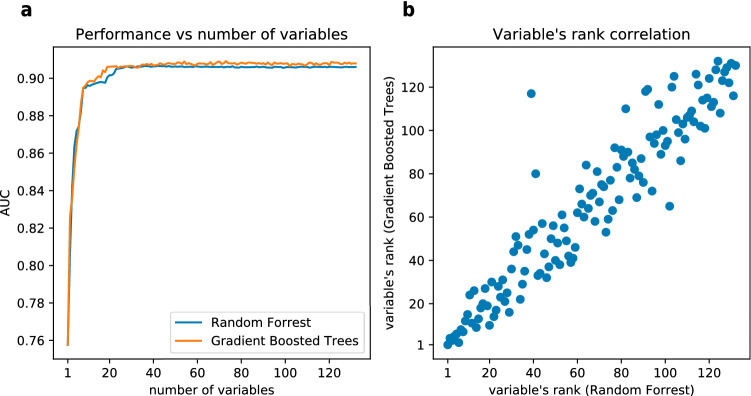
Comparison between iterative variable elimination performed with GBT and RF evaluated with the development set of 2014 data. Plot (**a**) shows AUC measured over varying number of input variables, where for every number of variables the model was retrained and evaluated separately. Plot (**b**) shows correlation between rankings obtained by GBT and RF.

To identify which input variables made the largest contribution to the model, we conducted a variable importance analysis of the trained models. Analysing two models provides two independent rankings of input variables in the order of importance. Thus, allowing for a robust identification of the predictors as oppose to analysis of only one model. The two models with the highest AUC score on the development set, i.e. GBT and RF, were used separately within the iterative variable elimination meta-algorithm to produce the variable rankings. As shown in Fig. 3A, the predictive performance of the models decreased noticeably after limiting the number of input variables to less than the 25 most important ones. This trend is apparent for both GBT and RF. The top five variables were: the result of the current herd-level SICCT test, number of outgoing direct links to the farms that became bTB infected after the move (summed over 1440 days before the test), easting of the farm, northing of the farm, number of animals moved out of the farm over 30 days before the test. The next 20 variables were related to the past SICCT results and bTB breakdown history of farms, land cover data, animal births, animal movement and contact tracing. Importantly, no variable related to climate data was included in the 25 most important ones. Details on the list of input variables can be found in Supplementary Table 1.

The ranks produced by the two methods were compared in order to check the consistency of the selection of the 25 best input variables. Figure 3B presents high correlation between the rankings obtained by GBT and RF, especially in the high importance range. Such a high correlation implies that the same variables were consistently selected as the most important ones.

## Discussion

The GBT predictive model achieved significantly increased HSe (by 6.3%-points) and HSp (by 1.8%-points) compared to the herd-level SICCT test on the 2015 data. Consequently, 297 herds that were classified as negative by the herd-level SICCT test, were identified as infected with bTB by the ML model. The increase in HSe was apparent in Wales, West England and North England. Improving the herd-level bTB diagnostic strategy is of high importance as it is necessary for an effective disease control programme. Accurate diagnostics inform issuing control measures, such as animal movement restrictions, which can mitigate further disease spread. This limits the number of secondary cases, ultimately leading to progressively decreasing number of infected herds. Numerous studies attempted to improve the animal-level bTB diagnostic by combining the SICCT with the gamma-interferon test^22^ or developing antibody detection assays^23^. Both of these approaches lead to more expensive and time-consuming strategies. The approach presented in this paper, however, offers a cost-effective solution to improving herd-level diagnostics by utilising data which are routinely collected.

In previous studies, known risk factors and data potentially related to bTB risk were used to inform models producing bTB risk maps^24^ or predicting recurring bTB breakdowns^25^. The results show that such data can be successfully used as input into the models predicting bTB risk. These models are, however, of limited use for the bTB control programme in GB. The risk maps lack granularity, i.e. they only identify areas of increased bTB risk instead of individual herds. It is costly to issue control measures area-wide, so herd-level predictions are preferred. The recurring breakdowns model predicts high risk herds, but it only applies to a small subset of all the herds. In contrast, this study is a first attempt to build a predictive model providing bTB breakdown predictions for every SICCT-tested herd in GB.

Our model was rigorously evaluated in a scenario as close to a real-life situation as possible. In this scenario, a model was to be built using only historical data prior to 31st December 2014. Then, the model was used to make bTB breakdown predictions for the herd-level SICCT tests conducted over 2015. The bTB surveillance data from 2012 to 2014 was used to train, optimise and select the predictive model. The trained model was, then, evaluated with bTB surveillance data from 2015. Importantly, these data had not been used previously either to train any model, to optimise the model’s settings or to select the most accurate model. The performance improvement was shown to be statistically significant compared to using the herd-level SICCT testing results alone to detect bTB breakdowns. The predictions of the model can aid issuing animal movement restrictions and inform targeting of undetected high-risk farms for follow-up. In the future, such a model can also serve as the backbone of a decision support tool helping veterinarians interpret the herd-level SICCT test results in the field.

Here, we used an extensive dataset of 139 variables to characterise farms that were tested during bTB surveillance activities in GB and enable the development of our ML model. Some of these variables exhibit correlation between each other. For example, variables related to climate, such as temperature and downward radiation, are highly correlated with one another, while percentage of woodland is negatively correlated with human population density. However, an ideal set of input variables for developing a ML model contains variables which experience weak or no correlation between each other, but all are correlated to the target output. In this sense, the dataset of 139 intercorrelated variables suffers from redundancy. To overcome such a limitation, we selected a subset of predictor variables yielding the same accuracy as using the full set of predictors. By doing so, we reduced our list of variables to 25 using the iterative variable elimination technique, while achieving similar level of predictive performance as the full model. This subset of variables included: farm location, between-farm animal movements, links to infected farms, breakdown history of farms, number of animals tested and land cover around the farm. Importantly, the model achieved the same level of predictive performance when all the variables related to climate data were removed. This is informative for the development of datasets in future studies on bTB breakdown prediction. Focusing on variables which were shown to be successful predictors can help decrease the amount of time needed for pre-processing of data and training of ML models.

The spatial distribution of farms correctly predicted as bTB-positive in 2015 shows that the benefit of using the ML model was only apparent in Wales, West England and North England. These regions are characterised by their higher bTB incidence and more frequent testing regime than the rest of GB. For comparison, there were more than 8000 breakdowns in West England between 2012 and 2014, 672 breakdowns in East England but only 43 breakdowns in Scotland in the same period. The low and high bTB incidence regions of GB may be different from each other in terms of bTB spread dynamics and risk factors. This can cause a model trained on all regions to perform suboptimally for the low incidence regions (East England and Scotland). An alternative approach is to train a ML algorithm on low incidence regions only, which would result in a model specialised in these regions. The dataset from East England and Scotland, however, includes less than 1.3% positives and it has insufficient size to train a ML algorithm. The spatial imbalance in the whole dataset was, therefore, inevitable and inferences from our model need to be made cautiously. All ML methods used in this study produce a continuous estimate of breakdown probability (from 0 to 1), so the balance between HSe and HSp can be adjusted by changing the value of threshold (Fig. 1). If higher HSe is required, it can be increased at the expense of HSp. Hence, the GBT model can predict breakdowns in East England and Scotland with HSe better than herd-level SICCT test, but it would then yield worse HSp.

According to the case definition, the ground truth positives in the dataset were derived from the results of inspections for lesions and *M. bovis* culture which has limited sensitivity. It is possible that some of the infected animals were identified as bTB-negative by the lesion inspections, which led to herds incorrectly labelled as ground truth negatives in the dataset. Due to the lack of a perfect bTB diagnostic test, these mislabelled data instances were inevitable, therefore limiting our ability to accurately and robustly validate our predictions of breakdowns. For this reason, together with the fact that the animal-level specificity of the SICCT test in GB is estimated as 99.98%, it would be more conservative to treat reactors as bTB-positive irrespectively of the model’s output when implementing the ML model in a real-world situation. However, a model’s prediction can improve the interpretation of the herd-level SICCT test when its result is negative. In this case, movement restrictions and follow-up testing should be issued if a herd is classified as a future breakdown by the model.

In conclusion, our ML model obtained significantly higher HSe and HSp than the herd-level SICCT test alone. This shows that ML methods can work as a framework for incorporating risk factor data from multiple sources into a predictive model to improve early bTB detection. Using routinely collected and freely available data, we are now able to provide a data-driven guidance for herd-level bTB diagnostics. This can directly improve the bTB control programme in GB by producing a list of priority high-risk farms for routine SICCT testing, helping scheduling follow-up tests and imposing animal movement restrictions. Importantly, these additional control measures can mitigate further disease spread and limit the number of secondary cases. The ML-based sophisticated interpretation of the herd-level SICCT test proposed in this study can support policy-makers and it encourages better-informed decision making.

## Methods

### Data sources and input variables

In GB, it is compulsory to record whether each animal-level SICCT test is positive or negative. The records are gathered by the Animal and Plant Health Agency (APHA) into the bTB surveillance database (SAM) where every holding is uniquely identifiable by a County Parish Holding (CPH) number. The same holding identifiers are used in the Cattle Tracing System (CTS) database^26^, which tracks between-farm cattle movements in GB. Based on the CPH number, the two databases were combined and variables related to animal movements and SICCT tests were derived to provide input into the ML algorithms. These variables were compiled into a dataset where each data instance represented a herd at the time of the routine SICCT testing. In general, every available characteristic of a farm was expressed as a numeric value, where real numbers corresponded to continuous values (e.g., easting and northing of a herd) and 0 or 1 encoding corresponded to categorical variables (e.g., SICCT test interpretation basis—standard or severe).

### SICCT test results

Every record of a SICCT test result conducted in England, Wales and Scotland between 2012 and 2015 was used in this analysis, together with the test interpretation basis (standard or severe), date the test was carried out, the CPH number of the tested holding, herd size and whether a lesion inspection and/or *M. bovis* culture confirmed the infection. All animal-level test results were aggregated and dichotomised at the herd-level, informing whether at least one reactor had been found in a herd. In total, over 500,000 herd-level results were recorded during the study period. Variables capturing the bTB breakdown history of a farm were derived from records of the previous tests. Here, the farms were described by the number of days from the last three SICCT tests and their herd-level results, number of past bTB breakdowns, and number of days from the last three bTB breakdowns which were confirmed with lesions and the duration of these breakdowns in days.

### Between-farm cattle movement

The CTS database totals 160 million individual level movements between 2001 and 2016. This data was aggregated to provide the number of births, deaths and incoming and outgoing movements calculated within periods of 30, 60 and 90 days prior to a SICCT test. The sums were divided into direct movements and movements of animals which went through a market. Contact tracing was performed with the *EpiContactTrace* R package^27^ to calculate the number of chains linking the farm in question with bTB-infected herds. Here, a herd is defined as bTB-infected from 90 days prior to its confirmed breakdown. The chains were divided into direct chains of length one and indirect chains which linked two herds via other herds. The input variables describing the farm in question extracted in this analysis were the numbers of movements from the bTB-infected herds to the farm (incoming chains) and movements from the farm to the infected herds (outgoing chains) accumulated over periods of 30, 60, 90, 180 days and 1, 2, 4 years prior to the SICCT test. Furthermore, a variable specifying whether a farm was a dairy, beef or a mixed farm was included in the dataset.

### Location data

SAM and CTS use the CPH numbers of the holdings, therefore it is possible to infer geographical locations of the farms (northing and easting) and relate them to other types of data. The dataset was extended with data related to climate, land cover and human population, which can be indirectly linked to variability of the SICCT test sensitivity or the survival of the pathogen in environment. Robinson et al*.*^28^ compiled the Climate Hydrology and Ecology Research Support System meteorology dataset for Great Britain (CHESS-met), which consists of meteorological variables for the period between 1961 and 2015. The data has a map structure which is formatted as a 1 km resolution grid containing daily mean measures of humidity, precipitation, air pressure, downward longwave radiation, downward shortwave radiation, wind speed, temperature and estimated evapotranspiration. Each value was averaged over periods of 30 days and 1 year before a SICCT test. The Land Cover Map 2015^29^ presents a classification of GB land into 10 categories: broadleaf woodland, coniferous woodland, arable land, improved grassland, semi-natural grassland, mountain or heath, saltwater, freshwater, coastal, built-up areas or gardens. The percentage of each of the classes of land cover within a radius of 1, 5 and 25 km from the farm was calculated. UK Gridded Population 2011 based on Census 2011 and Land Cover Map 2015^30^ provided human population density within a radius of 1, 5 and 25 km from the farm.

### Machine learning

A ML method may be interpreted as a multivariate parameterised mathematical function taking a vector of features of a farm as an input and producing a classification of the farm as either bTB-free or future breakdown. The goal is to adjust the internal parameters, such that the output of the function matches the correct classification of the farms. The learning refers to optimisation of these parameters during a training phase according to a training dataset, which is a subset of data with known categories of instances. Once the parameters are set, the function can classify unseen data. In this study, four ML algorithms (Neural Network, Random Forest, Gradient Boosted Trees and Support Vector Classifier) were used and their performances were compared. All the methods were implemented in the Scikit Learn package of Python^31^.

### Data split

The whole dataset of herd-level SICCT test results extended with data related to bTB infection risk was divided into training, development and testing disjoint subsets by year. The training set included data from 2012 to 2013, totalling 246,298 data instances, 3.78% of which were ground truth positive. The development set included 128,768 data points from 2014, where 3.74% of data were positive. The testing set of 2015 consisted of 130,945 data instances, 3.58% of which were positive. For training a ML model, ideally, a training set contains equal numbers of positives and negatives, so that the trained model is unbiased towards any of the classes^32^. However, due to bTB-negative herds being more frequent than positive ones, the data instances within the training set were reweighted. Every positive instance was associated with weight ${w}_{p}$ as shown in Eq. (1). The weight of every negative instance was set to ${w}_{n}$ as shown in Eq. (2). In this way, the cumulative impact of all positive herds on the loss function was as large as for all the negative herds.

1 $$\begin{array}{*{20}c} {w_{p} = 1/p} \\ \end{array}$$

where p is the number of positives in the training set

2 $${w}_{n}=1/n$$

where $n$ equals the number of negatives in the training set.

### Hyperparameter optimization

Settings of the ML methods were adjusted iteratively according to their performance on the development set consisting of data from 2014. Grid search and random search were used to optimise the hyperparameters. The former algorithm exhaustively evaluates all the combinations of hyperparameter values within a specified range and step size. The latter search randomly drew values from specified distributions and kept track of the best-performing ones. The two optimisation methods obtained equivalent sets of hyperparameters leading to almost identical performance of the predictive models, however, the random search converged faster. The models were evaluated based on the area under the receiver operating characteristic curve (AUC), which is a Se-Sp dependence curve. In general, models with AUC of 0.5 can be interpreted as performing similarly to a random guess, models with AUC larger than 0.7 obtain good performance while those with AUC above 0.9 are excellent predictors. The AUC was also used to select the best-performing ML method.

### Variable importance

GBT and RF both provide levels of variable importance, referred to as mean decrease of gini impurity (MDG), calculated for every input variable. The details of importance calculation are given in the Scikit-learn package documentation^33^. The variable importance can be intuitively understood as a measure of how much the predictive performance of the model decreases when a specific input variable is removed. A larger decrease in performance implies higher importance of the variable. MDG on its own is not regarded as a robust importance metric, especially if data is intercorrelated i.e., input variables are correlated with each other. In that case, the ranking of input variables in the order of their importance can significantly change after one variable is removed. Iterative variable elimination (IVE), which is a meta-algorithm using MDG as a base importance metric, resolves this issue. IVE iteratively removes the least important input variables one by one, retrains the model and recomputes the MDG variable importance ranking at every step. In this manner, the final variable ranking, which is produced progressing from the least important to the most important variable, is robust to data intercorrelation. IVE was used in this study to obtain the input variable rankings for GBT and RF models.

### Decision threshold optimization

All four ML methods used in this study produced a continuous output value between zero and one, where zero corresponds to bTB-free herd and one refers to a future breakdown. A larger value corresponds to a higher level of confidence that a farm will experience a breakdown. In the real-world application of a model, a binary result (positive or negative) is required. The output can be dichotomised by a decision threshold, such that all values below the threshold are considered negative and the values equal or larger than the threshold give positive results. There is a trade-off between HSe and HSp; too low a threshold leads to high HSe sacrificing HSp, and too high a threshold decreases HSe while improving HSp. An acceptable level of HSp is context-dependent and in the case of bTB testing it can be assumed to be equal to the HSp of the herd-level SICCT test. In this study, the observed HSe and HSp of the herd-level SICCT test were calculated for the development set of 2014 data. These values were then used to optimise the decision threshold of the model. A threshold value causing the HSe of the model to be equal to the HSe of the herd-level SICCT test was found using the grid search. This threshold value corresponds to the HSp which is higher than the HSp of the herd-level SICCT test. A second threshold value leading to a HSp which is equal to the HSp of the herd-level SICCT test was found too. In this case, the HSe of the model was higher than the HSe of the herd-level SICCT test. The final decision threshold of the predictive model was set to the mean of the two threshold values and resulted in the HSe and HSp, which were both higher comparing to the herd-level SICCT test alone. The threshold value optimised with 2014 development data was also used in the testing phase.

### Hypothesis testing

To test whether the performance of the best ML model was significantly better than the herd-level SICCT test alone, both their HSes and HSps were compared. A paired samples Student’s t-test was applied to the distributions of HSe and HSp to test if the mean HSe of the ML model was significantly larger than the mean HSe of the herd-level SICCT test, and whether the mean HSp of the model was larger than the mean HSp of the herd-level SICCT test. The distributions were obtained by randomly splitting the testing set of 2015 data into 100 disjoint subsets. Then, the breakdown predictions were made for each of the subsets, and the HSe and HSp were calculated for the herd-level SICCT test and for the ML predictions separately in every testing subset. The HSe distribution of the ML model was compared to the HSe distribution of the herd-level SICCT test and the HSp distribution of the model was compared to the HSp distribution of the herd-level SICCT test to provide the corresponding probability values (*p*-values). The distributions were also used to calculate confidence intervals (CIs) of mean HSe and HSp obtained by the herd-level SICCT test and the best ML model.

## Supplementary Information

Supplementary Tables.

## Data Availability

Land Cover Map 2015 and CHESS meteorology dataset used in this study are available in the UK Centre for Ecology & Hydrology repository, https://eip.ceh.ac.uk/data. SICCT test results and cattle movement data analysed during this study are available in the SAM system and the Cattle Tracing System (CTS), respectively. These data are available only with permission of Department for Environment, Food, and Rural Affairs (DEFRA) – Animal and Plant Health Agency (APHA).

## Code availability

The computer code used in this study is available from the authors upon request.
