# Supplementary material for: Using machine learning improves predictions of herd-level bovine tuberculosis breakdowns in Great Britain
Source: Sci Rep. 2021 Jan 26;11:2208. doi: 10.1038/s41598-021-81716-4 (PMC7838174; doi:10.1038/s41598-021-81716-4)
Supplement: Supplementary file 1 — Supplementary Tables. [file 41598_2021_81716_MOESM1_ESM.pdf]

Using machine learning improves predictions of herd-level bovine tuberculosis breakdowns in Great Britain

K. Stański[1], S. Lycett[1], T. Porphyre[2] & B.M.de C. Bronsvoort[1]

[1] The Epidemiology, Economics and Risk Assessment (EERA) Group, The Roslin Institute, Royal (Dick) School of Veterinary Studies, University of Edinburgh, Easter Bush, Midlothian, United Kingdom

[2] Université de Lyon, Université Lyon 1, CNRS, VetAgro Sup, Laboratoire de Biométrie et Biologie Evolutive, Villeurbanne Cedex, France

Supplementary material

Supplementary Table 1. List of 25 most important input variables in the order of importance produced by the iterative variable elimination with Gradient Boosted Trees.

Variable Rank	Description of variable
1	Binary result of the herd-level SICCT test
2	Number of farms where animals with bTB lesions were found after cattle had been moved to them directly from the farm of interest, summed over the 1440 days prior to the test.
3	Farm location (easting)
4	Farm location (northing)
5	Number of animals moved out of the farm during the 30 days before the test
6	Number of days from the last bTB breakdown at the farm before the test
7	Number of animals tested
8	0/1 indication whether the severe interpretation was applied
9	Number of farms where animals with bTB lesions were found after cattle had been moved from them directly to the farm of interest, summed over the 1440 days prior to the test.
10	Number of animals born on the farm during the 60 days before the test
11	Percentage of land cover which is classified as arable calculated up to 100 km away from the farm
12	Percentage of land cover which is classified as coniferous woodland calculated up to 100 km away from the farm
13	Number of animals moved out of the farm during the 90 days before the test
14	Number of animals not tested during this testing session
15	Number of animals moved onto the farm during the 30 days before the test
16	Percentage of land cover which is classified as broadleaf woodland calculated up to 25 km away from the farm
17	Binary result of the last herd-level SICCT test before the test of interest

18	Number of farms where animals with bTB lesions were found after cattle had been moved to them directly from the farm of interest, summed over the 720 days prior to the test.
19	Binary result of the second last herd-level SICCT skin test before the test of interest
20	Percentage of land cover which is classified as improved grassland calculated up to 100 km away from the farm
21	Number of animals born on the farm during the 90 days before the test
22	Percentage of land cover which is classified as coastal calculated up to 100 km away from the farm
23	Number of days from the last herd-level SICCT test before the test of interest
24	Percentage of land cover which is classified as mountain, heath or bog calculated up to 100 km away from the farm
25	Number of farms where animals with bTB lesions were found after cattle had been moved from them directly to the farm of interest, summed over the 720 days prior to the test.

Supplementary Table 2. List of 139 input variables offered to machine learning models. The order is arbitrary.

Description of variable
Date of a SICCT test
Year in which the SICCT test was conducted
Day of year of a SICCT test
Indication of whether a farm is in West England.
Indication of whether a farm is in North England.
Indication of whether a farm is in East England.
Indication of whether a farm is in Wales.
Indication of whether a farm is in Scotland.
Binary result of the herd-level SICCT tests
Indication of whether the severe interpretation was applied
Number of animals tested
Number of animals on the farm which were not tested.
Farm location (easting)
Farm location (northing)
Number of days passed from the most recent SICCT test.
Binary result of the most recent SICCT test.
Number of days passed from the 2nd most recent SICCT test.
Binary result of the 2nd most recent SICCT test.
Number of days passed from the 3rd most recent SICCT test.
Binary result of the 3rd most recent SICCT test.
Number of breakdowns the farm has experienced in the past.
Number of days passed from the most recent breakdown.
Duration of the most recent breakdown (in days).
Number of days passed from the 2nd most recent breakdown.
Duration of the 2nd most recent breakdown (in days).
Number of days passed from the 3rd most recent breakdown.
Duration of the 3rd most recent breakdown (in days).
Influx of animals within 30 days before the SICCT test.
Influx of animals which went through a market within 30 days.
Number of animals born within 30 days.

Influx of animals within 60 days.
Influx of animals which went through a market within 60 days.
Number of animals born within 60 days.
Influx of animals within 90 days.
Influx of animals which went through a market within 90 days.
Number of animals born within 90 days.
Number of animals moved out of the farm within 30 days.
Number of animals moved out of the farm through a market within 30 days.
Number of animals which have died within 30 days.
Number of animals moved out of the farm within 60 days.
Number of animals moved out of the farm through a market within 60 days.
Number of animals which have died within 60 days.
Number of animals moved out of the farm within 90 days.
Number of animals moved out of the farm through a market within 90 days.
Number of animals which have died within 90 days.
Indication of whether it is a beef farm.
Indication of whether it is a dairy farm.
Number of breakdown farms which moved cattle into the farm within 1440 days.
Number of breakdown farms which received cattle from the farm within 1440 days.
Number of breakdown farms which moved cattle into the farm within 720 days.
Number of breakdown farms which received cattle from the farm within 720 days.
Number of breakdown farms which moved cattle into the farm within 360 days.
Number of breakdown farms which received cattle from the farm within 360 days.
Indication of whether there were movements from breakdown farms to the farm within 360 days.
Indication of whether there were movements from the farm into the breakdown farms within 360 days.
Indication of whether there were direct movements from breakdown farms to the farm within 180 days.
Indication of whether there were indirect movements from breakdown farms to the farm within 180 days.
Indication of whether there were direct movements from the farm into the breakdown farms within 180 days.
Indication of whether there were indirect movements from the farm into the breakdown farms within 180 days.
Indication of whether there were direct movements from breakdown farms to the farm within 90 days.
Indication of whether there were indirect movements from breakdown farms to the farm within 90 days.
Indication of whether there were direct movements from the farm into the breakdown farms within 90 days.
Indication of whether there were indirect movements from the farm into the breakdown farms within 90 days.
Indication of whether there were direct movements from breakdown farms to the farm within 60 days.
Indication of whether there were indirect movements from breakdown farms to the farm within 60 days.
Indication of whether there were direct movements from the farm into the breakdown farms within 60 days.
Indication of whether there were indirect movements from the farm into the breakdown farms within 60 days.
Indication of whether there were direct movements from breakdown farms to the farm within 30 days.
Indication of whether there were indirect movements from breakdown farms to the farm within 30 days.
Indication of whether there were direct movements from the farm into the breakdown farms within 30 days.
Indication of whether there were indirect movements from the farm into the breakdown farms within 30 days.
Mean Humidity within 30 days
Mean Precipitation within 30 days
Mean Air pressure within 30 days
Mean Downward longwave radiation within 30 days
Mean Downward shortwave radiation within 30 days
Mean Wind speed within 30 days
Mean Temperature within 30 days
Mean Potential evapotranspiration within 30 days
Mean Potential evapotranspiration with interception correction within 30 days
Mean Humidity within 360 days
Mean Precipitation within 360 days
Mean Air pressure within 360 days
Mean Downward longwave radiation within 360 days
Mean Downward shortwave radiation within 360 days
Mean Wind speed within 360 days
Mean Temperature within 360 days
Mean Potential evapotranspiration within 360 days

Mean Potential evapotranspiration with interception correction within 360 days
Percentage of broadleaf woodland in an area up to 1km from the farm.
Percentage of coniferous woodland in an area up to 1km from the farm.
Percentage of arable land in an area up to 1km from the farm.
Percentage of improved grassland in an area up to 1km from the farm.
Percentage of seminatural grassland in an area up to 1km from the farm.
Percentage of mountain, heath or bog in an area up to 1km from the farm.
Percentage of saltwater in an area up to 1km from the farm.
Percentage of freshwater in an area up to 1km from the farm.
Percentage of coastal land in an area up to 1km from the farm.
Percentage of built-up areas and gardens in an area up to 1km from the farm.
Percentage of broadleaf woodland in an area up to 5km from the farm.
Percentage of coniferous woodland in an area up to 5km from the farm.
Percentage of arable land in an area up to 5km from the farm.
Percentage of improved grassland in an area up to 5km from the farm.
Percentage of seminatural grassland in an area up to 5km from the farm.
Percentage of mountain, heath or bog in an area up to 5km from the farm.
Percentage of saltwater in an area up to 5km from the farm.
Percentage of freshwater in an area up to 5km from the farm.
Percentage of coastal land in an area up to 5km from the farm.
Percentage of built-up areas and gardens in an area up to 5km from the farm.
Percentage of broadleaf woodland in an area up to 25km from the farm.
Percentage of coniferous woodland in an area up to 25km from the farm.
Percentage of arable land in an area up to 25km from the farm.
Percentage of improved grassland in an area up to 25km from the farm.
Percentage of seminatural grassland in an area up to 25km from the farm.
Percentage of mountain, heath or bog in an area up to 25km from the farm.
Percentage of saltwater in an area up to 25km from the farm.
Percentage of freshwater in an area up to 25km from the farm.
Percentage of coastal land in an area up to 25km from the farm.
Percentage of built-up areas and gardens in an area up to 25km from the farm.
Percentage of broadleaf woodland in an area up to 50km from the farm.
Percentage of coniferous woodland in an area up to 50km from the farm.
Percentage of arable land in an area up to 50km from the farm.
Percentage of improved grassland in an area up to 50km from the farm.
Percentage of seminatural grassland in an area up to 50km from the farm.
Percentage of mountain, heath or bog in an area up to 50km from the farm.
Percentage of saltwater in an area up to 50km from the farm.
Percentage of freshwater in an area up to 50km from the farm.
Percentage of coastal land in an area up to 50km from the farm.
Percentage of built-up areas and gardens in an area up to 50km from the farm.
Percentage of broadleaf woodland in an area up to 100km from the farm.
Percentage of coniferous woodland in an area up to 100km from the farm.
Percentage of arable land in an area up to 100km from the farm.
Percentage of improved grassland in an area up to 100km from the farm.
Percentage of seminatural grassland in an area up to 100km from the farm.
Percentage of mountain, heath or bog in an area up to 100km from the farm.
Percentage of saltwater in an area up to 100km from the farm.
Percentage of freshwater in an area up to 100km from the farm.
Percentage of coastal land in an area up to 100km from the farm.
Percentage of built-up areas and gardens in an area up to 100km from the farm.
